# New Insights into Ruling Out Internal Herniations After Laparoscopic Gastric Bypass on the Abdominal CT Scan: *The OPERATE study*

**DOI:** 10.1007/s11695-025-07715-w

**Published:** 2025-02-04

**Authors:** Marjolein R. A. Vink, Barbara A. Hutten, Nienke van Olst, Sterre C. P. de Vet, Max Nieuwdorp, Arnold W. van de Laar, Jeroen A. W. Tielbeek, Victor E. A. Gerdes

**Affiliations:** 1 Spaarne Gasthuis, Haarlem, Netherlands; 2 Amsterdam UMC Location AMC, Amsterdam, Netherlands

**Keywords:** Internal herniation, Computed tomography, Standardized protocol, Radiological signs

## Abstract

**Background:**

Internal herniation (IH) is a potentially life-threatening complication after gastric bypass. Accurate diagnosis of IH remains challenging. This study aims to validate the Eindhoven2020 (EHV20) scoring system for ruling out IH and seeks to improve its diagnostic accuracy through additional radiologic parameters.

**Methods:**

Patients participating in a prospective study on abdominal pain after gastric bypass surgery were selected if a CT scan was performed. CT scans were scored following the EHV20 scoring system containing ten signs of IH to confirm the individual and collective accuracy of these signs. Also, we evaluated the diagnostic value of additional radiologic parameters: delayed passage of contrast, dilated intestinal loops, and free fluid.

**Results:**

A total of 375 patients with abdominal pain were included. IH was confirmed during laparoscopy in 27 patients. On CT, the highest sensitivity was achieved by the swirl sign (66.7%) and the highest specificity by a small bowel behind the superior mesenteric artery (99.7%). The area under the receiver operating characteristic curve (AUC) based on the EHV20 scoring system for ruling out IH was 0.845 (95% CI 0.730–0.959). The AUC could be improved to 0.905 (95% CI 0.825–0.985) (*p* = 0.088) through the incorporation of several additional signs. Overall, this new scoring system included swirl sign, small bowel obstruction, enlarged nodes, venous congestion, mesenteric edema, dilated alimentary or biliary loop, free fluid, and backward flow in the biliary loop with possible backflow in the residual stomach.

**Conclusions:**

Incorporation of additional CT signs into an existing scoring system can help clinicians to safely rule out IH in patients with abdominal pain after bariatric surgery.

**Supplementary Information:**

The online version contains supplementary material available at 10.1007/s11695-025-07715-w.

## Introduction

Obesity is emerging as a major global public health concern. The increasing number of people suffering from severe obesity is leading to a growing demand for bariatric surgery (BS) [1–4]. Long-term follow-up has shown that acute and chronic abdominal pain are frequent and disabling complications after BS. The prevalence of both acute and chronic abdominal pain in patients with a gastric bypass (GBP) is estimated to be between 33.8 and 54.4% [5–7]. One of the causes of abdominal pain following GBP is internal herniation (IH). The incidence of IH is 2.5% [8]. IH can pose a life-threatening risk when it culminates into intestinal ischemia. Therefore, prompt and early detection of IH is of critical importance.

The gold standard in diagnosing IH is diagnostic laparoscopy. However, diagnostic laparoscopy is an invasive procedure. Since adequate tools to safely rule out IH in clinical practice are lacking, in many of these diagnostic laparoscopies, no IH is detected. Such unnecessary surgical procedures might be prevented by improved accuracy of computed tomography (CT) scanning. CT scans are commonly utilized in the diagnostic evaluation of IH, and their sensitivity and specificity have recently been improved. Nonetheless, the accuracy of CT scans in the diagnosis of IH remains a matter of debate [1–4, 9–12].

With the aim of achieving enhanced precision in abdominal CT scans for IH after BS, numerous studies have explored diverse distinctive CT signs of IH [1–4, 9, 10, 12, 13]. One of these studies by Ederveen et al. introduced a standardized scoring system for the assessment of the CT scan, containing the ten most frequently observed radiological signs of IH after laparoscopic Roux-en-Y Gastric Bypass [10]: swirl sign, small-bowel obstruction, clustered loops, mushroom sign, hurricane eye sign, small bowel behind the superior mesenteric artery (SMA), right-sided anastomosis, enlarged nodes, venous congestion, and mesenteric edema. A subsequent prospective study by the same study group demonstrated that this standardized scoring system for assessment of the CT scans (EHV20) improved the accuracy of abdominal CT scans in a clinical setting [1]. The implementation of a standardized scoring system is expected to improve the identification and safe exclusion of IH.

However, the EHV20 scoring system for IH has never been externally validated.

The aim of this study is to validate the standardized EHV20 scoring system in a bariatric cohort of patients with abdominal pain. In addition, we explore whether additional CT signs could enhance the diagnostic accuracy of the CT scan for diagnosing or excluding IH and whether specific combinations of signs can improve the model’s accuracy.

## Methods

### Study Population and Design

For this study, we selected patients from the OPERATE study, a prospective cohort study evaluating patients with a history of BS who present with abdominal pain at the Spaarne Gasthuis Hospital, a major bariatric center in the Netherlands. The OPERATE study started in December 2020, and the patient enrollment is still ongoing. All patients who sought care at the emergency department or the outpatient clinic due to abdominal pain complaints were included in the study. All participants received standard care. The study protocol was approved by the Local Ethics Committee. The principles of the Declaration of Helsinki were followed. Data collection procedure of this study has been previously described [14].

In the context of the current research, we included patients with a history of Roux-en-Y Gastric Bypass, a one anastomosis gastric bypass, or a revision surgery Roux-en-Y Gastric Bypass, as these bariatric procedures account for the vast majority of patients with a suspicion of IH. Patients who were subjected to a CT scan between December 2020 and April 2023 were included in this study. The study population consisted of both patients primarily operated at our hospital as well as those operated elsewhere. At our hospital, all Roux-en-Y Gastric Bypasses were performed using an antecolic antegastric alimentary limb approach. The surgical technique used in other hospitals is not documented for every patient. However, in the Netherlands, the antecolic antegastric alimentary limb approach is the standard procedure.

### Selection and Scoring of CT Scans and Reoperations

Standardized scoring systems were introduced in the routine clinical care management for patients with abdominal pain after BS encompassing both the assessment of the CT scans and the results of reoperations. For each patient, only the initial scan of an abdominal pain episode was included, even if multiple scans or pain episodes were available. An episode of pain was defined as the period from the first presentation at the hospital for abdominal pain until the day that the patient did not revisit the hospital for 3 consecutive months for abdominal pain. The rationale for including only the initial CT scan of each patient is to prevent a patient from being included twice in the study population. In the latter case, the observations will no longer be independent, which violates the assumptions of the statistical analyses used (logistic regression). In addition, the group of patients undergoing a repeat scan might have a different risk of IH at a repeat scan compared to the group of patients at a first scan and could therefore introduce bias. To avoid this bias and to fulfill the assumptions of the used statistical model, we chose to include only the first CT scan of a patient. Details of pain episodes in the OPERATE study have been described extensively previously [15]. CT scans performed without clinical suspicion of an IH were excluded. Whether a patient was clinically suspected of having an IH was based on the application for the abdominal CT scan filled out by the treating physician. Finally, we excluded scans that were not assessed according to the standardized scoring system. Figure 2 shows the flow diagram of the in- and exclusion criteria.

**Fig. 1:**
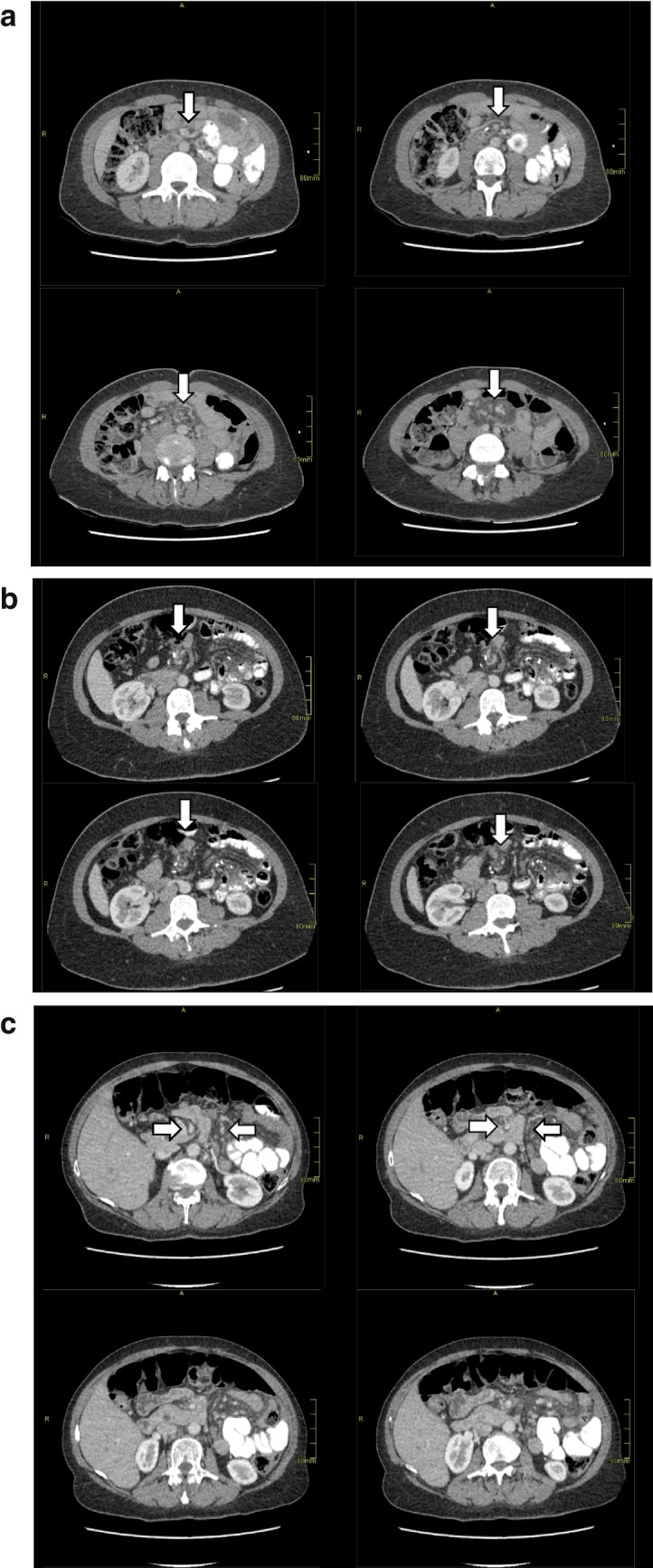
An example of **a** swirl sign and mesenteric edema in one patient from cranial to caudal, **b** a rotation that starts after the vena mesenteric superior from cranial to caudal which is typical for a hurricane eye sign, and **c** a herniated root via a surgical defect between the superior mesenteric artery and the distal mesenteric arterial branch with crowding and stretching of the mesentery from cranial to caudal which is typical for a mushroom sign

First, all abdominal CT scans were assessed for the ten most common signs of IH as described previously [1]: swirl sign, small bowel obstruction, clustered loops, mushroom sign (mushroom-shaped herniation of the mesenteric root), hurricane sign (rotation of the mesentery at a distal level with a tubular appearance of the distal mesenteric fat surrounded by bowel), small bowel behind the superior mesenteric artery (SMA), right-sided anastomosis, enlarged nodes, venous congestion, and mesenteric edema. For an example of a swirl sign, mushroom sign, and hurricane eye sign, see Fig. 1a–c. In addition to these signs, eight additional features were assessed: backflow of contrast in the biliary loop, backflow of contrast in the biliary loop and residual stomach, contrast that does not pass the jejunojejunal anastomosis, dilated alimentary loop, dilated biliary loop, dilated common channel, dilated residual stomach (differentiates where the small bowel obstruction is located), and free fluid. All CT scans were interpreted by the attending radiologist. We defined radiological suspicion of IH when the radiologist wrote in the concluding part of the CT scan report that there was suspicion of IH.

All reoperations performed in bariatric patients with abdominal pain in our hospital were included in the study database. Surgeons evaluated whether they observed open mesenteric defects, IH, and any complications such as ischemia or perforation of the intestines with the use of a standardized surgery report.

The diagnosis of IH was defined as bowel herniation through a mesenteric defect observed during diagnostic laparoscopy within 90 days after the abdominal CT scan. Absence of IH was defined as no bowel herniation was observed during surgery and a clinical negative follow-up of 90 days. This implies that within the 90-day period subsequent to the CT scan, neither a new CT scan was conducted nor the occurrence of a reoperation resulting in the identification of an IH occurred.

### Image Acquisition

CT scans were performed using Canon Aquilion ONE 320 and the Aquilion CXL 64 slice. Patient preparation consisted of drinking two cups of oral contrast 15 min before the start of the scan (Telebrix 300 mg/ml dissolved in 1000 ml) and one additional cup right before the start. In addition, according to contrast protocol, patients received Omnipaque 350 80 cc in combination with NaCl 0.9% 30 cc via intravenous access with a flow of 3 ml/sec. The CT scan contains a section thickness of 2.0 mm of the abdomen and pelvis in the portal venous phase of enhancement. Additional axial series with a slice thickness of 1.0 mm were reconstructed and added to the series. Rotation time of the scanners was 0.5 s and a fixed 120 kV technique.

### Data Collection

Patient characteristics at baseline were derived from electronic patient records. We registered the following variables: age, sex, weight, body mass index (BMI), and type of bariatric surgery.

All patient data underwent anonymization and were linked to a unique study number. Data collection was performed by three researchers, who were granted privileged access to the database. The data were gathered through a web-based data capture system Castor EDC.

### Statistical Analysis

Independent samples *t*-test (normally distributed variables) or the Mann–Whitney *U* test (skewed variables) were used for the comparison of continuous data between groups, and chi-square tests for categorical data. The sensitivity, specificity, positive predictive value (PPV), and negative predictive value (NPV) were derived through the analysis of two-way contingency tables.

Logistic regression models were used to assess the association between signs and IH. Additionally, we constructed receiver operating characteristic (ROC) curves and calculated the area under the curve (AUC). We developed a final model by stepwise evaluating each possible combination of the signs; the final model had the highest AUC. The AUCs obtained using the EHV20 scoring system and our scoring system, which includes the additional signs, were compared using the DeLong test to assess whether there was a statistically significant difference in their performances.

Statistical analyses were performed with SPSS software version 24.0 (SPSS Inc, Chicago, Ill).

## Results

### Description of the Study Population

**Fig. 2:**
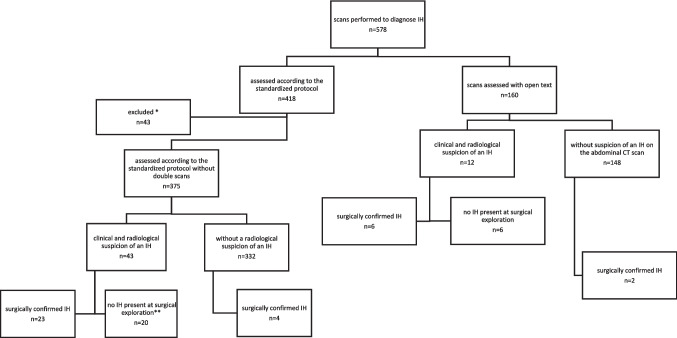
Flow diagram for the patient inclusion for this study. *Excluded because of double episodes or consecutive CT scans in one episode. **Surgical findings: 1 × intussusception, 1 × necrotic cholecystitis, 1 × jejunal stenosis, 1 × gastrojejunal stenosis, 4 × adhesive small bowel obstruction, 1 × perforated gastrojejunal anastomosis perforation, 11 × no abnormalities found. IH, internal herniation

In total, 375 patients were eligible for analysis. Patient characteristics are listed in Table 1. The evaluation of 375 scans of these patients led to a suspicion of an IH in 43 cases, assessed by the attending radiologists. In 23 cases, IH was present during surgical exploration (Fig. 2). In four patients with IH, there was no radiologic suspicion of IH. One of these IH was found during cholecystectomy, others were identified during diagnostic laparoscopy performed due to a high clinical suspicion of IH, despite the absence of radiological evidence of IH. In all four cases, there were distended loops but without signs of ischemia. Groups of patients with and without IH were largely comparable, although patients with IH were more often male (25.9% vs 7.8%, *p* = 0.002).

**Table 1:** Demographic and clinical characteristics of the study population

	**Total patient group** ***n* = 375**	**Internal herniation** ***n* = 27**	**No internal herniation** ***n* = 348**	***p*-value**
Sex – *n* (%)				0.002
Male	34 (9.1)	7 (25.9)	27 (7.8)	
Female	341 (90.9)	20 (74.1)	321 (92.2)	
Age (years) – mean (SD)	45.6 (12.1)	45.0 (11.8)	45.6 (12.1)	0.438
BMI before surgery (kg/m^2^) – median (IQR)	41.6 (38.8–45.3)	42.3 (37.7–44.8)	41.6 (38.8–45.5)	0.969
Time between surgery and CT-scan (months) – median (IQR)	42.0 (13.0–74.0)	52.0 (18.0–88.0)	41.0 (12.0–71.8)	0.277
Internal herniation in the past requiring surgery – *n* (%)	41 (10.9)	3 (7.3)	24 (7.2)	0.975
Abdominal surgery in the past – *n* (%)	206 (54.9)	15 (7.3)	12 (7.1)	0.946
**Comorbidities** – ***n* (%)**				
Type 2 diabetes				0.742
Insulin dependent	7 (1.9)	1 (3.7)	6 (1.7)	
Non-insulin dependent	24 (6.4)	2 (7.4)	22 (6.3)	
Hypertension	66 (17.6)	3 (11.1)	63 (18.1)	0.358
Obstructive sleep apnea	55 (14.7)	4 (15.4)	51 (14.7)	0.982
Dyslipidemia	29 (7.7)	4 (15.4)	25 (7.2)	0.153
Psychological disorders	100 (26.7)	5 (19.2)	95 (27.3)	0.370

*BMI* body mass index, *IQR* interquartile range,* n* number, *SD* standard deviation.

### Sensitivity and Specificity Rates

Table 2 shows the sensitivity, specificity, positive predictive value (PPV), and negative predictive value (NPV) of the different potential signs of IH. Previously described signs of swirl sign, mesenteric edema, and venous congestion have the highest sensitivity in our cohort, respectively 66.7%, 55.6%, and 44.4%. The newly added sign “free fluid” ranks 4th with 37.0% sensitivity. The highest specificity is observed for small bowel behind SMA (99.7%), swirl sign (98.9%), and mesenteric edema (98.9%).

**Table 2:** Sensitivity, specificity, positive and negative predictive values of the signs of internal herniation

	**Sensitivity** **% (95% CI)**	**Specificity** **% (95% CI)**	**PPV** **% (95% CI)**	**NPV** **% (95% CI)**
10 signs of Ederveen (10)				
Swirl sign	66.7 (48.0–82.4)	98.9 (97.4–99.6)	81.8 (62.7–94.0)	97.5 (95.5–98.8)
Small bowel obstruction	29.6 (14.8–48.2)	95.1 (92.5–97.1)	32.0 (16.1–51.4)	94.6 (91.9–96.6)
Clustered loops	18.5 (7.1–35.7)	95.1 (92.5–97.1)	22.7 (8.8–42.6)	93.8 (90.9–96.0)
Mushroom sign	11.1 (2.9–26.3)	99.4 (98.2–99.9)	60.0 (19.9–91.9)	93.5 (90.7–95.7)
Hurricane eye sign	22.2 (9.5–40.0)	98.6 (96.9–99.5)	54.5 (26.5–80.6)	94.2 (91.5–96.3)
Small bowel behind SMA	7.4 (1.3–21.2)	99.7 (98.7–100.0)	66.7 (16.1–97.7)	93.3 (90.4–95.5)
Right-sided anastomosis	7.4 (1.3–21.2)	100.0 (100.0–100.0)	100.0 (100.0–100.0)	93.3 (90.5–95.5)
Enlarged nodes	14.8 (4.9–31.1)	96.3 (93.9–97.9)	23.5 (8.0–46.5)	93.6 (90.7–95.8)
Venous congestion	44.4 (26.9–63.0)	98.3 (96.5–99.3)	66.7 (43.7–85.2)	95.8 (93.4–97.6)
Mesenteric edema	55.6 (37.0–73.1)	98.9 (97.4–99.6)	78.9 (57.6–92.9)	96.6 (94.4–98.2)
Additional signs				
Backflow of contrast in the biliary loop	3.8 (0.2–15.9)	94.2 (91.4–96.3)	4.8 (0.3–19.3)	92.8 (89.8–95.2)
Backflow of contrast in the biliary loop and residual stomach	0.0 (0.0–0.0)	97.7 (95.7–98.9)	0.0 (0.0–0.0)	92.8 (89.8–95.2)
Contrast does not pass the JJ-anastomosis	15.4 (5.1–32.2)	99.1 (97.7–99.8)	57.1 (22.7–87.1)	93.9 (91.1–96.1)
Dilated residual stomach	3.7 (0.2–15.3)	98.0 (96.1–99.1)	12.5 (0.8–44.5)	92.9 (89.9–95.2)
Dilated alimentary loop	22.2 (9.5–40.0)	98.0 (96.1–99.1)	46.2 (21.6–72.1)	94.2 (91.4–96.3)
Dilated biliary loop	7.4 (1.3–21.2)	98.6 (96.9–99.5)	28.6 (5.4–65.0)	93.2 (90.3–95.5)
Dilated common channel	0.0 (0.0–0.0)	98.8 (97.3–99.6)	0.0 (0.0–0.0)	92.7 (89.7–95.0)
Free fluid	37.0 (20.6–55.8)	89.9 (86.5–92.8)	22.2 (11.8–35.7)	94.8 (92.1–96.9)

*CI* confidence interval, *NA* not applicable, *NPV* negative predictive value, *PPV* positive predictive value

**Fig. 3:**
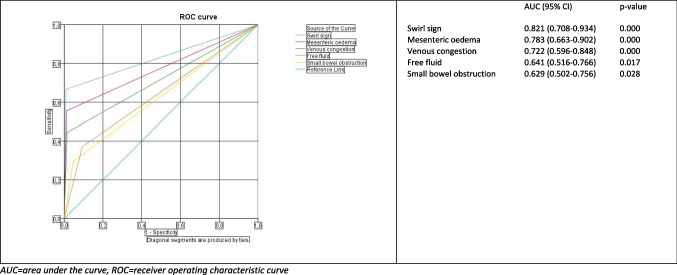
Receiver operating characteristic curves for clinical signs predicting internal herniation after bariatric surgery. AUC, area under the curve; ROC, receiver operating characteristic curve

Figure 3 shows the ROC curves and AUCs of these signs. The swirl sign has the highest AUC (0.821), followed by mesenteric edema (0.783), venous congestion (0.722), and free fluid (0.641). The AUC of dilated common channel and dilated residual stomach values are comparable to the reference line in our study.

### Existing Versus Optimal Model

**Fig. 4:**
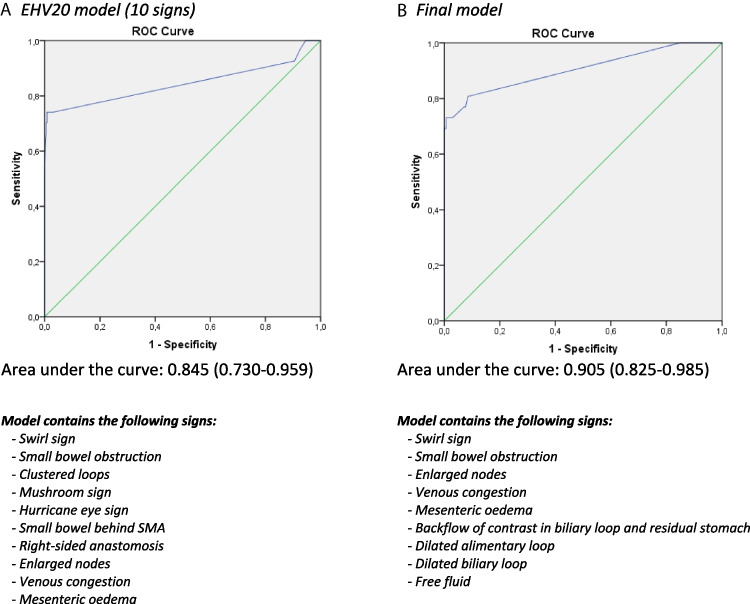
Comparison of the EHV20 model and the final model

The observed AUC of the EHV 20 scoring system with ten signs was 0.845 (95% CI 0.730–0.959). In a model in which all additional signs were added, the AUC was 0.892 (95% CI 0.802–0.982). In the most optimal model which used relevant signs only, we observed an AUC of 0.905 (95% CI 0.825–0.985) (Fig. 4). The difference in AUC between the EHV20 model and the final model did not reach statistical significance (*p* = 0.088). The optimal model with the highest AUC in our study contains five signs of the previously described EHV20 scoring system (swirl sign, small bowel obstruction, enlarged nodes, venous congestion, and mesenteric edema) and five additional signs (dilated alimentary loop, dilated biliary loop, free fluid, backward flow in the biliary loop, and residual stomach).

## Discussion

In this study, we validated a standardized system of scoring CT images using ten signs of IH by radiologists in clinical practice. Utilizing this standardized system facilitated relatively clear differentiation between patients with and without IH (AUC of 0.845). Furthermore, we demonstrated additional signs associated with IH: dilated alimentary loop, the dilated biliary loop, free fluid, the backward flow in the biliary loop, and the backward flow of contrast in the biliary loop in combination with the backflow of contrast into the residual stomach (Table 2). Among these examined radiological signs, free fluid was actually significantly associated with IH. In addition, we demonstrated that a new improved scoring system may be more effective, although direct comparison did not reach statistical significance. We used five signs of the EHV20 scoring system (swirl sign, small bowel obstruction, enlarged nodes, venous congestion, mesenteric edema) together with five additional signs to develop a new scoring system to discern the presence or absence of an IH. For implementation in clinical practice, prospective validation of the improved new scoring system is needed.

In 2020, Ederveen et al. analyzed 174 CT scans and diagnosed 32 people (18%) with an IH [1]. This percentage of IH is considerably higher than the 7% found in our study. A possible explanation for this difference could be that our study included patients both with acute abdominal pain at the emergency department and at the outpatient clinic, whereas Ederveen likely only included patients with acute abdominal pain at the emergency department, but this exact description is lacking. In addition, the patients in Ederveen’s study were included from 2011 to 2017, and they only started closing mesenteric windows in that center from 2017 onwards. Studies indicate that the incidence of IH is higher in patients where the mesenteric windows were not primarily closed [16]. Furthermore, a study by Giudicelli et al. evaluated not only CT scans but also body temperature and laboratory results in four separate small cohorts [13]. In line with our results, Giudicelli et al. described that free fluid on a CT scan provided an important indication of IH. Overall, when combining body temperature, laboratory and CT results their scoring system with IH as outcome variable had an AUC of 0.846 (95% CI: 0.78–0.91), whereas our discrimination model solely based on CT showed a higher AUC.

The sensitivity and specificity of various signs that may indicate an IH have been previously investigated. In our study, we showed that the swirl sign had the highest sensitivity of all evaluated signs (66.7%). Swirl sign has been described previously with a sensitivity ranging between 61 and 100% [1, 3, 4, 9, 10, 12, 17, 18]. In addition, the swirl sign showed the highest positive predictive value in our study (81.8 (CI 62.7–94.0)). The free fluid sign has previously been described as a good indicator of IH in only one study [13]. Giudicelli et al. described in this study whether the free fluid was visible in one, two, three, or four quadrants. In that study, free fluid was only visible in one quadrant in most cases. We did not specify the location of the fluid in our study, as we believe this will not further improve diagnostic accuracy for the presence or absence of IH. Interestingly, we found a difference with Ederveen’s study regarding the sensitivity in the mushroom and hurricane eye signs [10]. Whereas Ederveen et al. showed a sensitivity of 54.3% and 51.4%, respectively, we found low sensitivities of 11.1% and 22.2%. One potential explanation could be that the radiologists in Ederveen et al. fully reassessed the CT scans for the study and were therefore specifically focused on the ten study signs. In contrast, we used the evaluation of CT scans in daily clinical practice. This may suggest that these signs often remain unnoticed in routine clinical practice. This emphasized the importance of the development of a scoring system that not merely focuses on statistical accuracy but is also easy to use and reproducible in daily practice, especially for novice or non-abdominal specialized radiologists. The scoring system used in this study has already been implemented in daily practice for more than 3 years in our hospital and is used by radiologists for the vast majority of CT evaluations with IH suspicion. Nevertheless, at times, due to workload or other reasons, adherence to the scoring system was not optimal, as observed with the exclusion of the 160 scans without a standardized scoring system. However, overall, the scoring system is commonly utilized and with our validation, it can be regarded as a tool to systematically assess CT scans for the presence or absence of IH.

Despite the swirl sign demonstrating a sensitivity of 60%, the remaining signs exhibited relatively low individual sensitivity. This implies that the evaluation and combination of multiple signs are required to reliably ascertain the presence of IH. The newly described signs in our scoring system help this determination, with a sensitivity of 37.0% for free fluid and 22.2% for a dilated alimentary loop. However, due to the low incidence of IH in patients with abdominal pain, obtaining high specificity in our scoring system is of the most added value in daily clinical practice. Each diagnostic indicator exhibited an individual specificity between 89.9 and 99.7%, indicative of a high degree of confidence in the exclusion of IH. This certainty is particularly strong when signs are considered collectively, as demonstrated by our model’s findings.

Our study has several strengths. The sample size of our study is large compared to previously published work. We included 375 scans for analysis and excluded paired scans, scans that were not performed because of clinical suspicion of an IH, and those that were not scored according to the standardized scoring system. This provides a comprehensive representation of clinical practice. Prior studies included all CT scans from individual patients, leading to analytical noise arising from duplicated patient inclusions. In addition, our study is prospective, whereas many studies investigate the radiological signs of IH retrospectively.

Furthermore, our study has several limitations. First, all CT scans were interpreted by the attending radiologist. This induces inter-observer variability in CT evaluation. In our hospital, no distinction is made between different radiology specialties during weekend, evening, or night shifts. This may reduce the quality of the assessment of the CT scan when it is assessed by a general radiologist rather than a specialized abdominal radiologist. Previous studies have already investigated inter-observer differences. However, these inter-observer differences are beyond the scope of this study, which focused on the overall assessments in daily practice. On the other hand, it is also a strength of our study because this study directly reflects daily practice. Second, all signs of IH were binary scored rather than with open text fields to facilitate statistical analysis of the collected information and the building of predictive models. However, this may have resulted in the loss of nuances. Considerable or little free fluid, minimally distended or severely distended intestinal loops, and mildly or hugely enlarged lymph nodes: all these are examples of indicators of IH on scans that received binary scores. As a final limitation, one could discuss that in our study, all bariatric surgeries were performed with an antecolic approach, while in some countries, the retrocolic approach is also used. In a subsequent study, it is imperative to conduct further investigation to ascertain whether the enhanced accuracy of the scoring system extends to patients with a history of bariatric surgery employing a retrocolic approach.

## Conclusion

We validated the existing EHV20 scoring system for the evaluation of IH in patients with abdominal pain after bariatric surgery. In addition, our improved scoring system suggested improvement in ruling out IH on CT scans, but the comparison with the existing EHV20 scoring system did not reach statistical significance (*p* = 0.088). Further research should attempt to enhance the diagnostic value of current and additional CT signs and our scoring system even further, with the aim of minimizing unnecessary diagnostic laparoscopies in future clinical practice.

## Supplementary Information

Below is the link to the electronic supplementary material.

Supplementary file1 (DOCX 77 KB)

## Data Availability

No datasets were generated or analysed during the current study.
