# Supplementary material for: New Insights into Ruling Out Internal Herniations After Laparoscopic Gastric Bypass on the Abdominal CT Scan: The OPERATE study
Source: Obes Surg. 2025 Feb 4;35(3):715–24. doi: 10.1007/s11695-025-07715-w (PMC11906499; doi:10.1007/s11695-025-07715-w)
Supplement: Supplementary file 1 — Supplementary file1 (DOCX 77 KB) [file 11695_2025_7715_MOESM1_ESM.docx]

**Supplementary Information**

*Supplemental figure 1: Area under the receiver operating characteristic curve for all clinical signs predicting internal herniation after bariatric surgery*

| 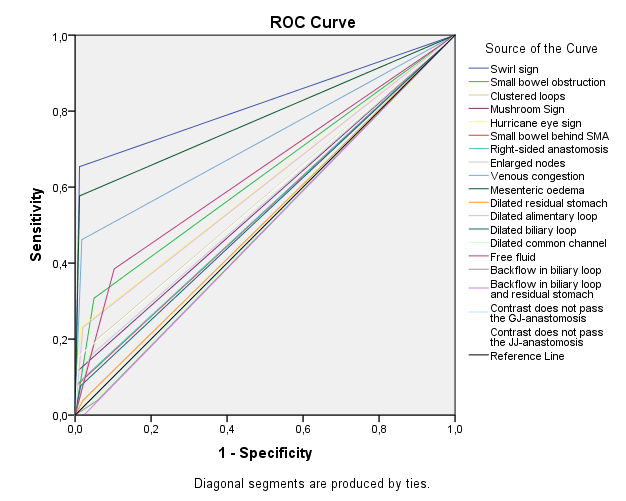 | \|  \| AUC (95% CI) \| p-value \| \| --- \| --- \| --- \| \| Swirl sign \| 0.821 (0.708-0.934) \| 0.000 \| \| Mesenteric oedema \| 0.783 (0.663-0.902) \| 0.000 \| \| Venous congestion \| 0.722 (0.596-0.848) \| 0.000 \| \| Free fluid \| 0.641 (0.516-0.766) \| 0.017 \| \| Small bowel obstruction \| 0.629 (0.502-0.756) \| 0.028 \| \| Hurricane eye sign \| 0.608 (0.480-0.736) \| 0.066 \| \| Dilated alimentary loop \| 0.605 (0.478-0.733) \| 0.074 \| \| Contrast does not pass the JJ-anastomosis \| 0.573 (0.447-0.698) \| 0.218 \| \| Clustered loops \| 0.571 (0.447-0.696) \| 0.226 \| \| Enlarged nodes \| 0.558 (0.434-0.681) \| 0.325 \| \| Mushroom Sign \| 0.555 (0.431-0.679) \| 0.352 \| \| Right-sided anastomosis \| 0.538 (0.417-0.660) \| 0.513 \| \| Small bowel behind SMA \| 0.537 (0.415-0.658) \| 0.529 \| \| Dilated biliary loop \| 0.531 (0.411-0.652) \| 0.597 \| \| Dilated residual stomach \| 0.509 (0.392-0.626) \| 0.879 \| \| Contrast does not pass the GJ-anastomosis \| 0.500 (0.385-0.615) \| 1.000 \| \| Dilated common channel \| 0.494 (0.380-0.608) \| 0.921 \| \| Backflow in biliary loop \| 0.490 (0.376-0.603) \| 0.864 \| \| Backflow in biliary loop and residual stomach \| 0.488 (0.375-0.601) \| 0.842 \| |
| --- | --- | --- | --- | --- | --- | --- | --- | --- | --- | --- | --- | --- | --- | --- | --- | --- | --- | --- | --- | --- | --- | --- | --- | --- | --- | --- | --- | --- | --- | --- | --- | --- | --- | --- | --- | --- | --- | --- | --- | --- | --- | --- | --- | --- | --- | --- | --- | --- | --- | --- | --- | --- | --- | --- | --- | --- | --- | --- | --- | --- | --- |

*AUC=area under the curve, ROC=receiver operating characteristic curve, SMA=superior mesenteric artery*
